# Brain activation and heart rate variability as markers of autonomic function under stress

**DOI:** 10.1038/s41598-025-12430-8

**Published:** 2025-08-01

**Authors:** Annika Huber, Julian Koenig, Bastian Bruns, Martin Bendszus, Hans-Christoph Friederich, Joe J. Simon

**Affiliations:** 1 Department of General Internal Medicine and Psychosomatics, Centre for Psychosocial Medicine, University Hospital Heidelberg, Im Neuenheimer Feld 410, 69120 Heidelberg, Germany; 2 Department of Child and Adolescent Psychiatry, Psychosomatics and Psychotherapy, University Hospital Cologne, Faculty of Medicine, University of Cologne, Cologne, Germany; 3 Department of Cardiology, Angiology and Pneumology, University Hospital Heidelberg, Heidelberg, Germany; 4 Department of Neuroradiology, University Hospital Heidelberg, Heidelberg, Germany; 5 DZPG (German Centre for Mental Health – Partner Site Heidelberg/ Mannheim/ Ulm), Heidelberg, Germany

**Keywords:** Heart rate variability, Stress, Functional MRI, Central autonomic network, Generalized additive modelling, Psychology, Stress and resilience

## Abstract

Efficient brain–heart interactions, mediated by the central autonomic network (CAN), are crucial in regulating physiological and psychological stress. The ability of the autonomic nervous system to adapt to stress predicts resilience to cardiovascular, anxiety, and mood disorders. Since the neural dynamics underlying brain–heart interactions remain poorly understood, this study investigated brain activation and heart rate variability (HRV) during stress and relaxation. Functional magnetic resonance imaging (fMRI) and peripheral heart rate assessment were used to assess brain–heart coupling during breathing-induced relaxation, psychosocial stress and stress recovery in 32 healthy participants. We assessed the relation between perceived stress and brain activation, and employed non-linear generalized additive models to forecast changes in HR based on brain activation in the CAN. Both breathing-induced relaxation and stress induction significantly affected HR variation and triggered brain activation in CAN-related regions. HR variation was related to CAN activity during stress induction, and that chronic stress was linked to reduced brain activation during stress recovery. Finally, we demonstrated that brain activation within the CAN predicts changes in HRV. Our results offer novel insights into dynamic brain–heart interactions during stress-related autonomic regulation and emphasize the brain–heart axis’s potential as a target for therapeutic interventions aimed at enhancing stress resilience.

## Introduction

A healthy organism is characterized by its ability to adapt to changing internal and external circumstances, such as temperature fluctuations, increased mental demands, or pain. This adaptability is partly achieved through autonomic regulation, mediated by the parasympathetic and sympathetic nervous systems, as well as receptor arcs and humoral responses, which work together to adjust functions like heart rate and blood pressure^1^. Chronic and acute stress have been linked to dysfunctional autonomic regulation, which has been shown to have a profound negative impact on overall health. A reduced physiological adaptability to stress has been linked to cardiovascular diseases such as myocardial ischemia^2^ or hypertension^3^, but also to psychiatric disorders such anxiety disorders and depression^4,5^.

The body and the brain are interconnected by dynamic structural and functional networks, including the central autonomic network (CAN^6^). It is an integral component of an internal regulation system through which the brain controls cardiovascular and other autonomic responses such as response to stress as well as emotion regulation ^7^. While autonomic control through the CAN is exerted via both the sympathetic and parasympathetic nervous system, at rest the parasympathetic nervous system is the dominant effector of autonomic control. Thus, the CAN directly influences cardiac vagal activity and heart rate variability (HRV^8^). The CAN includes a number of regions identified as key in autonomic regulation^7^. For example, the anterior cingulate cortex (ACC) is essential for evaluating threats and safety, modulating autonomic arousal to meet behavioral demands during a stress response^9,10^. Furthermore, parts of the insula^11^ and the ventral medial prefrontal cortex^12^, as well as subcortical regions, including the thalamus^13^, amygdala and cerebellum^14^, have been shown to display increased activation in conjunction with parasympathetic cardiovascular activity, suggesting their significance in influencing efferent vagal activity. While the present work focuses on top-down modulation of brain–heart coupling, there is increasing research supporting the notion of significant bottom-up influences from the heart and other visceral signals to the brain. Bottom-up signaling from the heart and gut in order for the brain to maintain predictive models of bodily needs and maintain interoception are essential, shaping the brain’s top-down responses^9,15^. Furthermore, heart rate oscillations have been shown to directly strengthen medial prefrontal regulatory regions of the brain in particular, influencing emotion regulation^16^, with some studies even suggesting heart rate oscillations (measured through HRV) to have a stronger effect on neural activation than the other way around^17^. These findings reflect the bidirectional pathways of heart-brain interaction, whose exact coupling remains largely unknown.

An essential marker of autonomic flexibility is HRV, defined as the variation in beat-to-beat intervals (IBI). Autonomic, cardiovascular, and respiratory systems all exert their influence on HRV and produce short term changes in HRV, particularly through the parasympathetic nervous system^18^, which exhibits tonic inhibitory control over influences from the sympathetic nervous system^19^. It has been shown that parasympathetic influences take effect quickly (in the realm of milliseconds) versus seconds in the case of sympathetic effectors^20^. This temporal difference is presumably due to the short effect latency as well as higher turnover rate of acetylcholine (as the parasympathetic effector), compared to norepinephrine (as the sympathetic effector)^21^. This means that short-term fluctuation in HRV can be attributed to vagal control, highlighting HRV as a marker of short-term autonomic flexibility^22^. High HRV is indicative of intact autonomic adaptability of an organism while a reduced HRV is associated with a vulnerability to physical and psychological stressors^23^ and is commonly observed in disorders characterized by autonomic dysregulation, such as depression and anxiety^8^ or posttraumatic stress disorder^2^.

Previous studies have highlighted the role of HRV as an indicator of brain–heart interaction^6^. Specifically, reduced HRV is associated with increased sympathetic drive and enhanced activation in brain regions associated with autonomic and emotional regulation, such as the ACC, insula and periaqueductal gray^9,24^. Deep (slow) breathing at frequencies of 5.5 to 6 breaths per minute has been shown to maximize parasympathetic cardiac regulation and HRV^16,25^, as well as increase activation in the CAN^13^. This is achieved through complex pathways, importantly the baroreceptor reflex. Due to the expansion of the lungs during inspiration, baroreceptors, which are sensitive to pressure, are stimulated in the aortic arch, causing heart rate to decrease^26^. The reverse effect occurs during expiration. This phenomenon is also known as respiratory sinus arrhythmia (RSA), which has been found to relate to high-frequency components of HRV, such as the root mean square of successive differences (RMSSD)^27^. Deep-breathing is already utilized in biofeedback interventions, which reliably increases HRV, mood, and autonomic adaptability^28^. Overall, deep-breathing techniques promote autonomic adjustments that improve HRV and respiratory sinus arrhythmia, accompanied by central nervous system changes that enhance autonomic, cerebral and psychological flexibility^29^. As such, HRV reflects the top-down influences on dynamic autonomic cardiorespiratory coupling^8^. This interplay underscores the CAN’s role in mediating autonomic flexibility and its potential as a therapeutic target in conditions marked by autonomic dysregulation, using deep-breathing as a facilitator for increased vagal control. However, while previous research has revealed close links between the CAN and autonomic regulation, critical gaps remain in understanding how HRV is modulated by higher cortical autonomic structures, particularly in response to stress.

Therefore, to gain a better understanding of the dynamic and complex relationship within the heart-brain axis, the aim of the current study is to explore neuronal brain–heart coupling related to stress resilience and autonomic adaptability. We investigated brain activation and peripheral measures of vagal activity during transitions between stress and relaxation states. By employing breathing-induced relaxation as well as psychosocial stress induction, we aimed to investigate the underlying mechanisms of neuronal autonomic control. We hypothesized that changes in HRV, especially during deep-breathing, will be associated with distinct brain activation patterns within the CAN. This may enhance our understanding of brain–heart interactions and its role in stress resilience, which presents an important extension to the existing literature.

## Methods

### Participants

We recruited 35 healthy participants through community advertisements. Inclusion criteria: right-handed, over 18, no mental illness history (screened using the Structured Clinical Interview for DSM-IV^30^, no neurological disorder or head injury, smoking or MRI contraindications. Three participants were excluded due to excessive head movement during scanning (> 4 mm, ± 3° of rotation), leading to a sample size of N = 32. Participants completed questionnaires assessing anxiety (STAI^31^), depression (BDI-II^32^), emotion regulation (DERS^33^, ERQ^34^), chronic stress (TICS-SSCS^35^), subjective perceived stress (PSS^36^) and verbal intelligence (MWT-B^37^). The study was approved by the ethics committee of the Medical Faculty of the University of Heidelberg (S-070/2022) and conducted in accordance with the Declaration of Helsinki. Participants provided written informed consent.

### Procedures

Each participant underwent a single MRI-scanning session. During MRI-scanning, participants performed a paced-breathing and stress induction task. They completed questionnaires before scanning, and the experimental task was explained.

### fMRI task

**Fig. 1:**
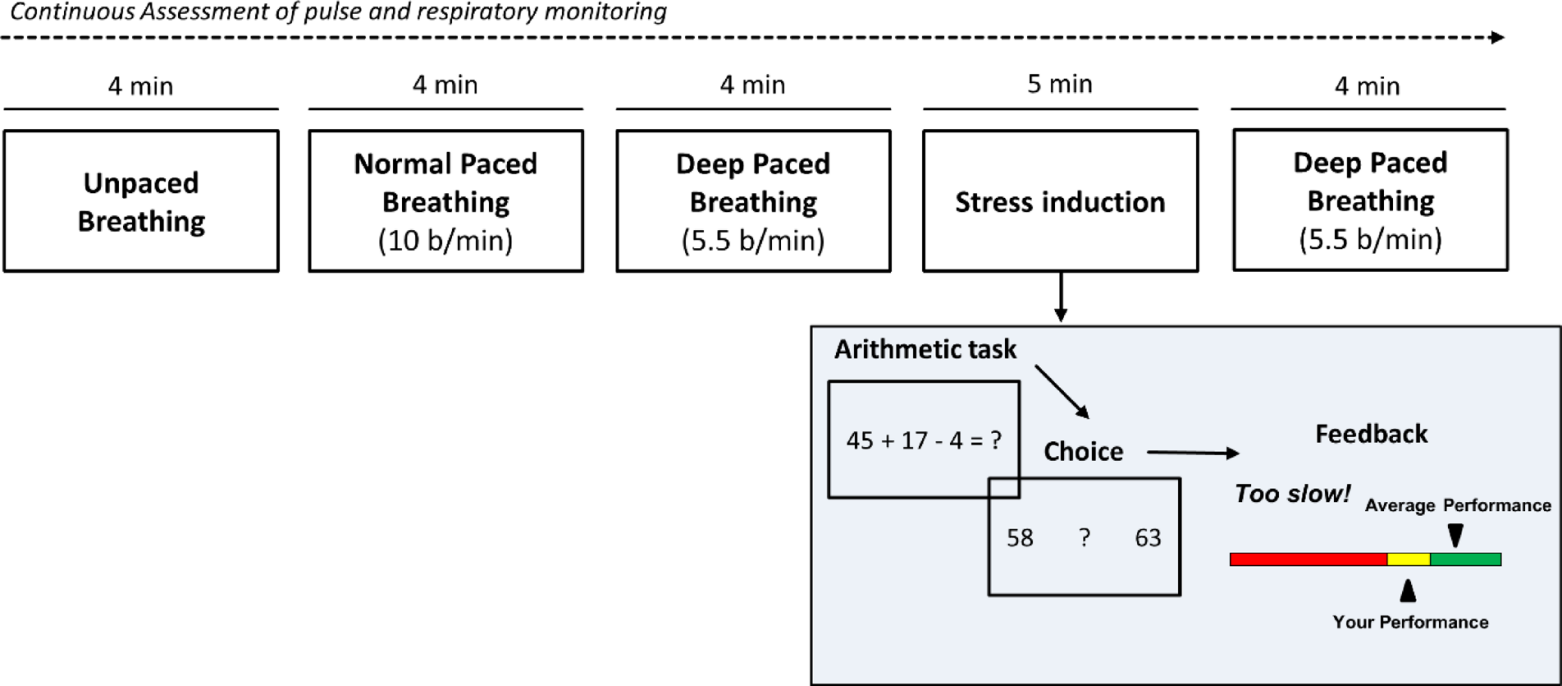
Graphical depiction of the Deep-Breathing stress-induction fMRI task. (**A**) The experimental task starts with 4 min of unpaced (free) breathing, followed by 4 min of paced breathing with an interval of 10 breaths per minute and 4 min of slow breathing (deep-paced breathing, 5.5 breaths per minute). Following this, participants had to complete a modified version of the Montreal Imaging Stress Task (Dedovic et al. 2005), where simple arithmetic problems have to be solved within a time-frame individually calculated to be too short to solve the task, and feedback regarding their own performance relative to the average performance of other participants was provided on a mock performance bar, manipulated to exaggerate the average performance. The task finished with a “recovery-from-stress” block of 4 min of slow breathing.

We employed a “deep-breathing” task combined with a well validated stress-induction paradigm^38^. Participants were instructed to follow a paced breathing exercise consisting of 5 blocks. The task begins with 4 min of normal breathing, where participants are free to breathe at their own pace (“unpaced breathing”). Following this, participants are instructed via a visual cue to breathe at a normal rate of 10 breaths/min for 4 min (“normal paced breathing”). This block serves as the control condition for the following block of deep-paced breathing (4 min with a breathing frequency of 5.5 breaths/min). A breathing frequency of ~ 6 breaths/min has been found to consistently increase HRV^29^. Following this, participants had to undergo the Montreal Imaging Stress Task (MIST^38^), which is a frequently employed fMRI stress paradigm that has be shown to reliably induce brain responses associated with psychosocial stress^39,40^. The task combines the stress-eliciting effects of high cognitive demand with negative social evaluation. Participants must solve simple arithmetic tasks (f.ex.: 145–17 + 3) and submit their response via a response button box. Each arithmetic problem has to be solved within a time period individually calculated to be just beyond the participant’s computing speed. Following each trial, participants receive feedback on the submitted response (“correct,” “incorrect” or “too slow”) as well as regarding their own average performance relative to the average performance of all other participants (which is artificially set to 80% success) on a mock performance bar. Following this, a “recovery-from-stress” block of 4 min of deep-breathing (5.5 breaths/min) was administered. Figure 1 provides a graphical depiction of the task.

Heart rate was continuously monitored during scanning using a photoplethysmography (PPG) sensor attached to the proximal phalanx of the left index finger (achieved sampling rate of 500 Hz). Local maxima of PPG signal were automatically detected, and timestamps of PPG peaks (P-peaks) were recorded by the MR Scanner. Respiration was continuously measured using inductive plethysmography via a respiratory cushion integrated into a belt fitted around the participant’s ribcage. The cushion was connected by a pressure hose to the ECG and respiratory sensor unit (PERU), which recorded respiration curves in real time. Both PPG and respiratory signals were acquired using built-in systems provided by the MRI vendor. Stimuli were presented using Presentation V18 (Neurobehavioral Systems, Berkeley, Calif., USA). The total duration of the task was 20 min.

### fMRI acquisition

Images were collected using a Prisma Fit 3-T whole-body MR scanner (Siemens Medical Solutions, Erlangen, Germany) equipped with a standard 32-channel head coil. 40 oblique interleaved slices were acquired parallel to the AC-PC axis using a T2*-sensitive single-shot EPI sequence with following parameters: TR = 2500 ms, TE = 26 ms, flip angle = 80°, field of view = 154 × 154 mm with a matrix size of 96 × 96, in-plane resolution of 2.3 × 2.3 × 3.4mm^3^. A total of 500 images were collected during one single run. For anatomical reference, high-resolution T1 MPRAGE anatomical images were acquired (176 slices, voxel size 1 × 1 × 1 mm, TR = 1900 ms, TE = 2.26 ms, 9° flip angle).

### Statistical analysis

#### Analysis of heart rate variability

The IBIs were calculated as the peak-to-peak interval of photoplethysmography signal (PPG). The quality of peak detection was manually inspected using an in-house Matlab script by overlapping scanner detected peak timestamps over PPG signal. Errors in peaks due to ectopic beats or participant movement were identified using a percentage filter (IBIs increase or decrease of more than 30% compared to the previous interval) and subsequently interpolated. The root mean square of the successive difference in adjacent IBIs (RMSSD), which allows to measure short-term variations of IBI, was calculated for each individual. This allows an individual assessment of HRV^6^. The RMSSD metric primarily reflects the high-frequency (parasympathetic) component of HRV^41^ and reliably reflects vagal activity^18,42,43^›, whereas the IBI closely associated with both parasympathetic and sympathetic activity^44^. To assess the association between HRV and the experimental task, in-house scripts in STATA (Version 18; StataCorp LP, College Station, TX, USA) were used to calculate mean values of IBI and RMSSD corresponding to each block of the task. To evaluate the effect of the breathing and stress tasks on IBI and RMSSD values, we employed a one-way repeated measures ANOVA using natural log-corrected (ln) IBI and RMSSD values. We employed natural log-corrected (ln) IBI and RMSSD values because heart rate variability measures typically exhibit positive skew and heteroscedasticity. Log transformation helps to normalize their distribution, reduce the impact of extreme values, and stabilize variance, thereby improving the reliability and interpretability of statistical analyses. This approach is standard in HRV research and has been recommended in previous methodological work to meet the assumptions of parametric models^45^.Post hoc comparisons of IBI and RMSSD values across different blocks were performed using Tukey’s Honestly Significant Difference (HSD) test. Analyses were performed using GraphPad Prism version 10.0.0 (GraphPad Software, Boston, Massachusetts USA). To assess the association between HRV and brain activation, we adopted a sliding window approach^46^ in STATA, where both IBI and RMSSD time-series were resampled at the fMRI TR and subsequently mean-centered. Each window had a length of 20 s and shifted according to the fMRI TR (2.5 s). We chose a duration of 20 s due to its ability to balance temporal resolution and physiological relevance. Previous studies have shown that this duration is sufficient for reliable HRV time-domain analysis, while maintaining responsiveness to dynamic changes in autonomic function ^47–50^. The resulting time-series were then used as a regressor in the general linear model (GLM) fMRI analysis (see below). This approach allows for the examination of temporal dynamics in physiological signals, capturing changes over time and relating them to brain activity. Finally, the relation between HRV and psychometric assessment of stress and anxiety was assessed in an exploratory analysis using the Pearson product-moment correlation coefficient (2-tailed, *P* < 0.05). Specifically, differences between the experimental conditions in IBI and RMSSD values were correlated with individual scores on stress perception (PSS and TICS-SCSS scales) and anxiety (STAI scale).

### fMRI analysis

Pre-processing and statistical analyses of fMRI data were conducted using SPM12 (Wellcome Trust, London, UK). The following pre-processing steps were used: Exclusion of first four volumes to account for magnetic field for equilibration, slice time correction, realignment and unwarping to correct for artifacts due to susceptibility-by-movement interactions, coregistration of T1 images with the mean T2* images, normalization to MNI space (functional: 3mm3, anatomical: 1mm3), smoothing of functional images (8 mm Gaussian kernel). Subsequently, a general linear model was constructed employing a block-design, where the different events during the task were modeled using boxcar functions. A 480 s high-pass filter was used to remove low-frequency noise and signal drift. The following individual contrast images corresponding to the effects of interest were constructed: a) “Relaxation or central autonomic network (CAN)” contrast (deep-paced breathing vs. normal-paced breathing), b) “Stress Reactivity” contrast (stress induction vs. deep-paced breathing), c) “Stress Recovery Specific Relaxation” contrast (recovery deep-paced breathing vs. deep-paced breathing) and d) “Post Stress Relaxation” contrast (recovery deep-paced breathing vs. normal-paced breathing). Furthermore, for purposes of masking and explicit baseline correction (see below), we computed contrasts for the comparison of stress induction vs. unpaced breathing and stress induction vs. normal-paced breathing. A fifth contrast coined “Stress Resiliency” (recovery deep-paced breathing vs. stress induction), was not included in the fMRI analysis, since the comparison between brain activation during paced breathing and the MIST task would suffer from reduced power, given the large-scale activation patterns observed during the MIST task. However, this contrast was used in the analysis of HRV data.

At the group level, we performed five different types of analyses. In a first step, we assessed brain activation related to the different blocks of the experimental task. One-sample T-tests were employed for each of the four contrasts of interest. To ensure that the reported activation patterns in the contrast “Stress Reactivity” (i.e., stress induction vs. deep-paced breathing) specifically reflect differences between stress and the relaxation response induced by deep-paced breathing, we employed exclusive masking. Specifically, we constructed masks based on activation observed in the contrasts “stress induction vs. unpaced breathing” and “stress induction vs. normal-paced breathing” (each with a cluster-defining voxel-wise threshold of family-wise error (FEW) corrected *P* < 0.0001). These contrasts were chosen because paced breathing in these conditions is not expected to elicit strong relaxation effects. By excluding shared activation between the stress condition and less relaxing breathing conditions, we aimed to isolate stress-related brain activity and eliminate non-specific effects related to visual stimulation or guided breathing. This exclusive masking approach was only applied to the “Stress Reactivity” contrast. For all contrasts of interests, whole-brain results are reported at a cluster-defining voxel-wise threshold of FWE-corrected *P* < 0.05 and minimal cluster size k > 10, significant results are reported at a cluster-level significance threshold of FWE-corrected *P* < 0.05. However, to achieve increased specificity of results, for the contrast “Stress Reactivity”, results are reported using an cluster-defining voxel-wise threshold of FWE-corrected *P* < 0.0001.

In a second step, we assessed the relation between HRV and fMRI activation during the experimental task. To this end, we computed two additional GLMs for each participant, one including a regressor for RMSSD values and one including a regressor for IBI values^13,51^. Both values were re-sampled at the fMRI TR using a sliding window approach. The influence of including HRV-related regressors in the GLM was assessed statistically by means of paired T-tests comparing the 1st level results of each subject with and without inclusion of regressors. Whole-brain results are reported at an uncorrected cluster-defining voxel-wise threshold of *P* < 0.001 and minimal cluster size k > 10, significant results are reported at a cluster-level FWE-corrected significance threshold of *P* < 0.05.

In a third step, we performed a whole-brain regression analysis to assess the relationship between task-induced brain activation and subjective perceived stress, assessed using the PSS and TICS-SCSS scales. For each of the four contrasts of interest, additional one-sample T-tests were performed including individual scale scores as regressors of interest. Results are reported at an uncorrected cluster-defining voxel-wise threshold of *P* < 0.001 and minimal cluster size k > 10, significant results are reported at a cluster-level FWE-corrected significance threshold of *P* < 0.05.

In a fourth step, we sought to assess the predictive relationship between brain activation and HRV. Given that the inclusion of a heart activity as a regressor in the first level GLM of individual participants only allows to test for a correlational relationship and does not allow to infer directionality, we adopted a complementary approach by using brain activation as the predictor of HRV measures. Since non-linear associations between neural activity and HRV are commonly observed^52,53^, we employed Generalized Additive Models (GAMs^54^), using the mgcv package in R. GAMs are an extension of generalized linear models that allow for flexible data-driven, non-parametric modeling of non-linear relationships between predictors and the response variable by fitting smooth functions to each predictor, making them particularly suitable for capturing complex physiological interactions^55^. This is especially useful when analyzing physiological coupling, where relationships between brain- and heart activity are often non-linear and may vary across time or individuals. GAMs are able to account for intra-subject correlation within repeated measures data through random effects or penalized smooth terms. Finally, GAMs enhance interpretability by decomposing the contributions of individual predictors, making it easier to understand how specific physiological variables influence one another. Our model included time series of brain activation from seven a-priori chosen ROIs (see below) as predictor variables to forecast RMSSD and IBI values. We included IBI and RMSSD time-series resampled at the fMRI TR and constructed a separate model for IBI and RMSSD values. Experimental condition, subjects, and time points were included as parametric terms to control for their confounding effects on RMSSD and IBI changes. In our GAM model, we applied thin plate regression splines to model non-linear effects between neural activation and HRV. Smoothing parameters were estimated using restricted maximum likelihood (REML). To assess model robustness, we tested various model specifications, including those with and without lagged neural predictors, and versions incorporating random effects for subjects and time points. Model selection was guided by the proportion of explained deviance, and diagnostic checks (including gam.check(), concurvity inspection, and residual analysis) confirmed the validity of the smooth fits. The final models used to generate the results and visualizations included brain activation time series from seven a priori defined ROIs (see below) as smooth terms, with subject and condition included as fixed effects. Based on existing studies investigating brain regions in the central autonomic control of heart rate as well as involved during slow breathing, we used bilateral masks of the thalamus, anterior insula, amygdala, periaqueductal gray, ventral medial prefrontal cortex, medial and anterior cingulate cortex^6,9,13,42,56^. Importantly, these ROIs were chosen a priori and not based on the results observed in the current study. All masks were taken from the SPM Neuromorphometrics atlas, except the mask for the periaqueductal gray, which is not included in this atlas. The PAG mask was instead obtained from the Keuken and Forstmann’s 7T atlas^57^, resliced in MNI space and binarized. In an additional exploratory step, we also repeated the GAM analyses using separate ROIs for the left and right anterior insula, instead of a single bilateral mask, to investigate potential hemispheric differences in insula involvement. The left and right anterior insula ROIs were derived from the corresponding regions in the SPM Neuromorphometrics atlas. The same model specification, covariates, and model selection approach were used as in the original bilateral analysis.

Finally, to assess the directionality of coupling between heart rate variability (HRV) and brain activity, we performed Granger causality (GC) analyses at the group level using the vars package in R^58^. For each participant, time series were extracted from each ROI as the first eigenvariate of the BOLD signal within the mask, then temporally aligned with HRV metrics (IBI and RMSSD) and averaged across subjects to obtain group-level time series per variable. No detrending, differencing, or scaling was applied to retain physiological signal characteristics. For each HRV–ROI pair, we fitted vector autoregressive (VAR) models, the optimal lag order for each bivariate model was determined using the Akaike Information Criterion (AIC). To evaluate statistical significance, we conducted a permutation-based null distribution. Specifically, 1000 permutations were generated by independently shuffling the HRV and ROI time series across time points. Empirical p-values were derived by comparing observed GC p-values to this null distribution and adjusted using Bonferroni correction. In addition to p-values, we report F-values as GC effect size estimates.

## Results

### Subject data

The demographic and clinical characteristics of participants are summarized in Table 1.

**Table 1:** Participant characteristics.

	Mean ± SD
Female/male	22/12 (N = 32)
Age	25.9 ± 3.2
BDI-II	5.3 ± 3.4
STAI—State	35 ± 6.4
STAI—Trait	36.4 ± 7.8
DERS Total score	66.7 ± 12.4
DERS Access	13.3 ± 3.8
DERS Nonacceptance	10.3 ± 3.3
DERS Goal-Directed	11.6 ± 3.6
DERS Impulse Control	8.6 ± 2.2
DERS Awareness	13.9 ± 4.1
DERS Clarity	8.9 ± 2.5
ERQ Cognitive Reappraisal	4.1 ± 0.7
ERQ Expressive Suppression	4.9 ± 0.8
TICS	16.4 ± 7.5
PSS	20 ± 5.8
MWT-B	30.6 ± 2.9

BDI-II, Beck Depression Inventory; STAI, State-Trait Anxiety Inventory; DERS, Difficulties in Emotion Regulation Scale; ERQ, Emotion Regulation Questionnaire; TICS, Trier Inventory for Chronic Stress; PSS, Perceived Stress Scale. MWT-B: Vocabulary-Based Test for the Assessment of Premorbid Intelligence.

### HRV findings

**Fig. 2:**
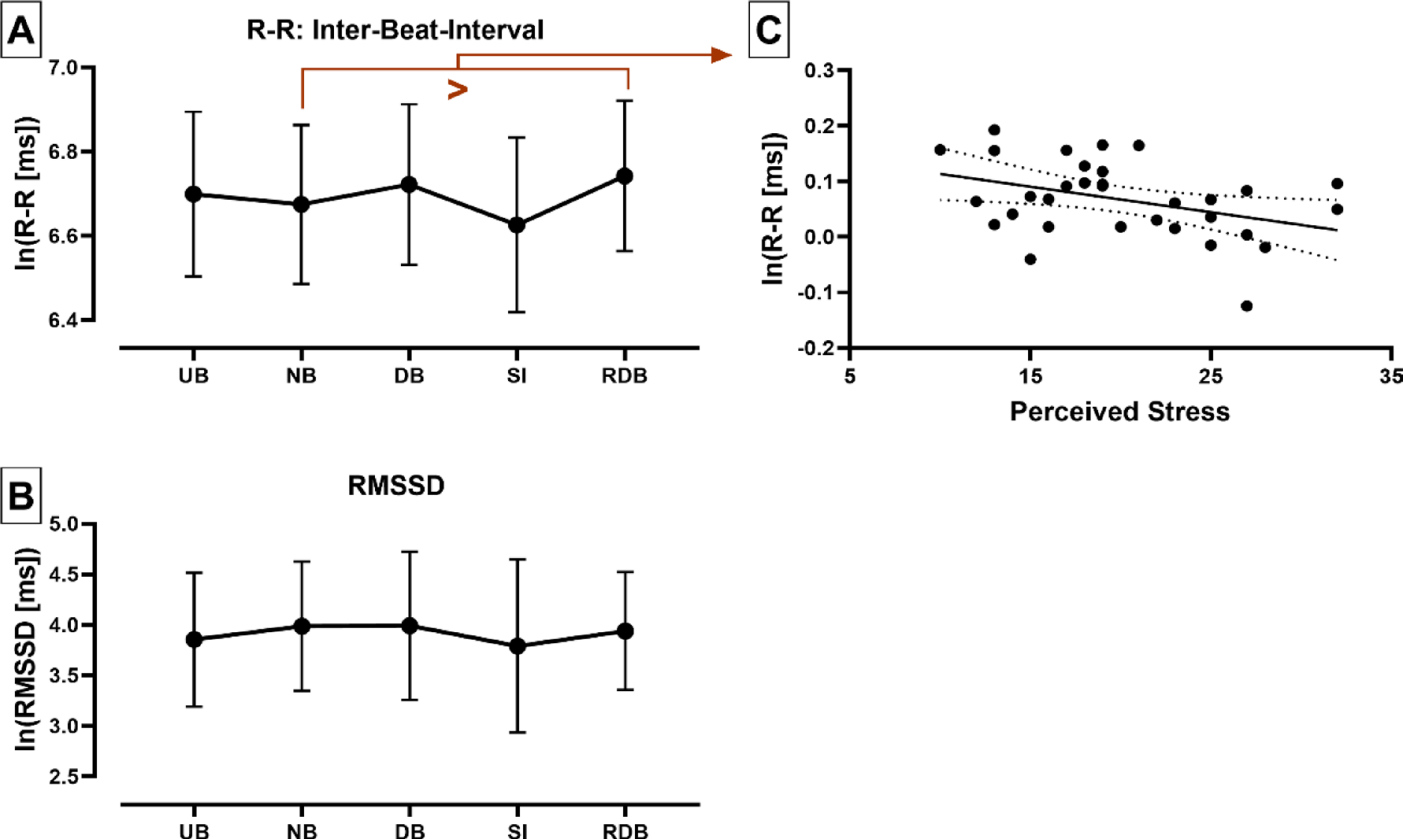
Changes in Heart Rate Variation during the experimental task. (**A**) Relation between the experimental task and peak-to-peak interval of photoplethysmography signal (inter-beat intervals, IBI, *P* = 0.0584, η^2^ = 0.08). UB = unpaced breathing, NB = normal-paced breathing, DB = deep (slow)-paced breathing, SI = Stress induction, RDB = Recovery deep(slow)-paced breathing. Sig. differences were observed between DB and NB (*P* < 0.001), SI and DB (*P* = 0.001), RDB and SI (*P* < 0.001) and RDB and NB (*P* < 0.001). (**B**) Relation between the experimental task and the root mean square of the successive difference in adjacent IBIs (RMSSD, non-sig.). (**C**) Negative correlation between mean scores of the “Perceived Stress Scale” and differences in Interbeat-Intervals when comparing recovery deep(slow)-paced breathing (RDB) with deep(slow)-paced breathing (DB, r = − 0.383, *P* = 0.0304). Importantly, this finding did not survive Bonferroni correction for multiple comparisons (20 comparisons per HRV metric: α = 0.0025). Pearson product-moment correlation coefficient (2-tailed). Regression line is depicted with 95% confidence bands.

We found a significant influence of experimental condition on IBI values (F_(2.491, 77.21)_ = 15.43, *P* < 0.001, η^2^ = 0.33), but not on RMSSD values (F_(2.402, 74.46)_ = 2.784, P = 0.0584, η^2^ = 0.08). Post-hoc Tests revealed a significant difference in IBI values between deep-paced breathing vs. normal-paced breathing (“Relaxation” contrast, Mean difference = 0.047, 95% CI [− 0.017, − 0.078], *P* < 0.001), stress induction vs. deep-paced breathing (“Stress Reactivity” contrast, Mean difference = − 0.096, 95% CI [0.159, 0.032], *P* = 0.001), recovery deep-paced breathing vs. stress induction (“Stress Resiliency” contrast, Mean difference = 0.116, 95% CI [− 0.061, − 0.171], *P* < 0.001) and recovery deep-paced breathing vs. normal-paced breathing (“Post Stress Relaxation” contrast, Mean difference = 0.067, 95% CI [− 0.032, − 0.103], *P* < 0.001). See Fig. 2a,b. We failed to observe any significant correlation between HRV values and mean scores of the STAI as well as TICS-SCSS scales, but we found a significant negative correlation between mean scores of the “Perceived Stress Scale” and differences in IBI when comparing recovery deep(slow)-paced breathing (RDB) with deep(slow)-paced breathing (DB, r = − 0.384, *P* = 0.0298). However, it must be noted that this result did not survive Bonferroni correction for multiple comparisons (20 comparisons per HRV metric: α = 0.0025, see Fig. 2c).

### fMRI Findings

#### Whole-brain fMRI activation during the experimental task

**Fig. 3:**
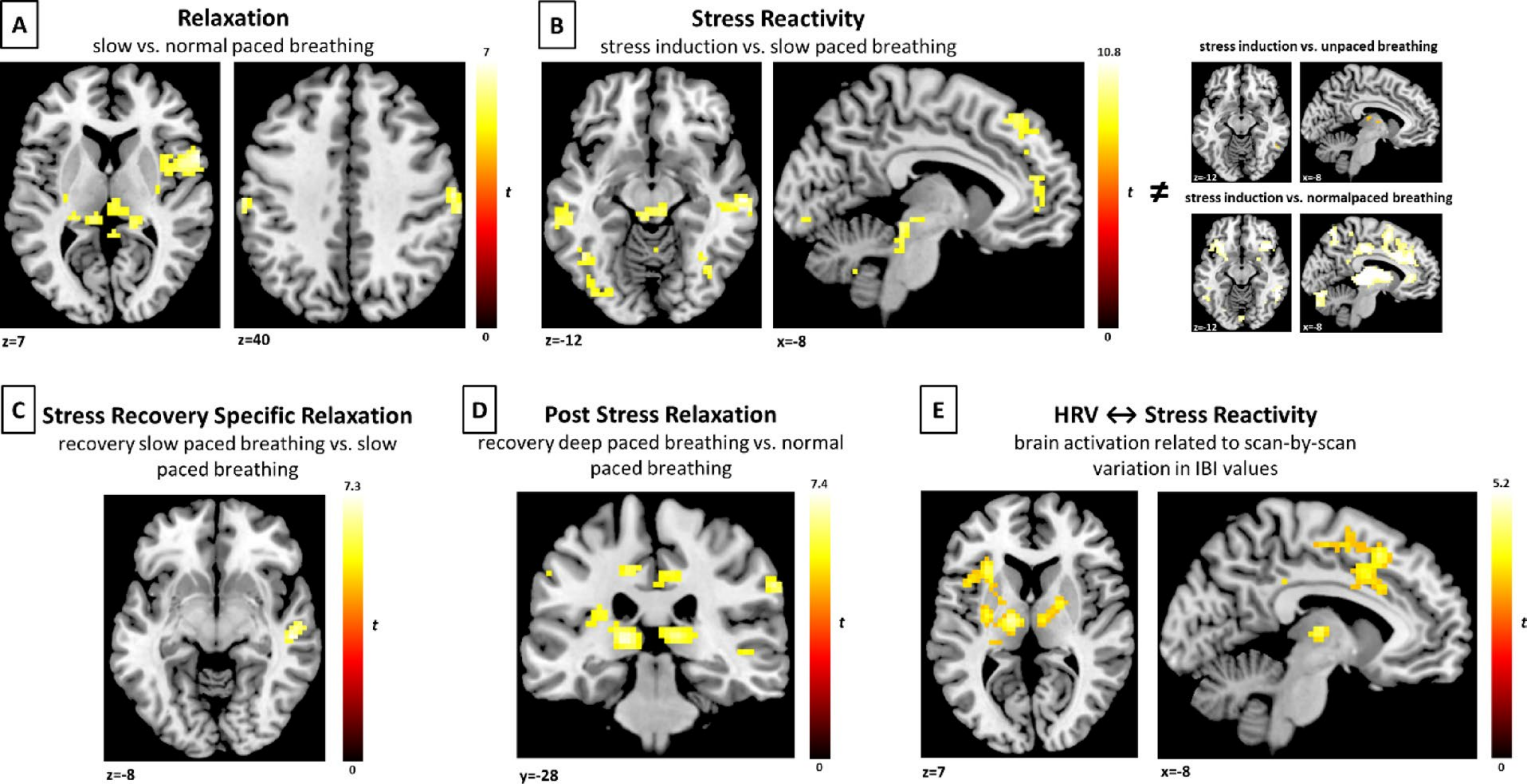
Whole-brain fMRI activation during paced breathing and stress induction. (**A**) Significant brain activation during the “Relaxation” contrast (deep-paced breathing vs. normal-paced breathing), shown here are activations in the thalamus and parietal operculum (left) and bilateral postcentral/somatosensory gyrus (right), (**B**) Significant brain activation during the “Stress Reactivity” contrast (stress induction vs. deep-paced breathing), activations in the periaqueductal gray, bilateral middle temporal gyrus (left) and anterior cingulate cortex (right) are shown. Results are exclusively masked with the contrasts “stress induction” vs. “unpaced breathing” and “stress induction” vs. “normal-paced breathing”, shown at the right. (**C**) “Stress Recovery Specific Relaxation” (recovery deep-paced breathing vs. deep-paced breathing), significant activation in the middle temporal gyrus is shown. (**D**) “Post Stress Relaxation” (recovery deep-paced breathing vs. normal-paced breathing), significant activations in the thalamus and medial cingulate gyrus are shown. (**E**) Influence of inter-beat intervals values on brain activation during the contrast “Stress Reactivity” (stress induction vs. deep-paced breathing), inclusion of IBI values (re-sampled at the fMRI TR) in individual first-level GLMs. Shown here are significant activations in the thalamus, putamen, anterior insula and medial cingulate. (**A**, **C**, **D**) All results significant at a cluster-level FWE-corrected significance threshold of *P* < 0.05, with a cluster defining threshold of FWE *P* < 0.05 and minimal cluster size k > 10. (**B**) Results significant at a cluster-level FWE-corrected significance threshold of P < 0.05, with a cluster-defining voxel-wise threshold of FWE *P* < 0.0001 and minimal cluster size k > 10. (**E**) All results significant at a cluster-level FWE-corrected significance threshold of *P* < 0.05, with a cluster-defining voxel-wise threshold of P < 0.001 uncorrected and minimal cluster size.

During the “Relaxation” contrast (deep-paced breathing vs. normal paced breathing), we observed significant activation in the bilateral postcentral/somatosensory gyrus, inferior frontal gyrus, thalamus and medial cingulate gyrus. During the “Stress Reactivity” contrast (stress induction vs. deep-paced breathing), we found activation in the middle and inferior temporal gyrus, occipital gyrus, periaqueductal gray, superior medial gyrus and anterior cingulate cortex. During “Stress Recovery Specific Relaxation” (recovery deep-paced breathing vs. deep-paced breathing), we observed activation in the middle temporal gyrus and during “Post Stress Relaxation” (recovery deep-paced breathing vs. normal paced breathing), we found significant activation in the thalamus, anterior insula, inferior parietal lobule, medial cingulate gyrus and precuneus (see Table 2 and Fig. 3).

**Table 2:** Whole-brain fMRI results during the relaxation—stress induction task.

Contrast/brain region	*P*-value	*k*	*t*-value	x	y	z
Relaxation—deep paced breathing vs. normal paced breathing						
Postcentral Gyrus/Somatosensory Gyrus	< 0.001	157	7.002	48	− 34	59
Parietal operculum	< 0.001	145	6.889	60	11	8
Thalamus	< 0.001	205	6.260	3	− 22	14
Postcentral Gyrus / Inferior Parietal Lobule	0.001	35	6.363	− 60	− 19	41
Medial Cingulate Gyrus	0.004	15	6.008	12	− 7	50
Stress Reactivity—stress induction vs. deep paced breathing						
Right Middle Temporal Gyrus	< 0.001	99	10.892	60	− 19	− 10
Left Middle Temporal Gyrus	< 0.001	52	10.617	− 60	− 31	− 7
Medial Occipitotemporal Gyrus	< 0.001	32	10.038	12	− 88	2
Inferior Temporal Gyrus	< 0.001	65	9.982	− 45	− 58	− 10
Inferior Occipital Gyrus	< 0.001	65	9.815	− 36	− 82	− 7
Brainstem / periaqueductal gray	< 0.001	70	9.686	− 3	− 31	− 16
Superior Medial Gyrus / Frontal Eye Field	< 0.001	85	9.432	12	38	53
Anterior Cingulate Cortex	< 0.001	39	9.287	− 3	50	14
Stress Recovery Specific Relaxation—recovery deep paced breathing vs. deep paced breathing						
Middle Temporal Gyrus	< 0.001	25	7.270	51	− 22	− 7
Post Stress Relaxation—recovery deep paced breathing vs. normal paced breathing						
Thalamus / Insula / Inferior Parietal Lobule	< 0.001	499	7.433	− 57	− 34	35
Superior Temporal Gyrus (WM)	< 0.001	152	7.107	45	− 49	20
Anterior Insula	< 0.001	47	6.758	36	23	− 10
Inferior Parietal Lobule	< 0.001	27	6.749	63	− 28	35
Thalamus	< 0.001	129	6.670	12	− 28	8
Left Medial Cingulate Gyrus	0.001	25	6.248	− 15	− 28	41
Right Medial Cingulate Gyrus	< 0.001	84	6.196	9	− 25	41
Precuneus	< 0.001	43	6.191	3	− 58	41

*k* = cluster size. Results significant at cluster-level P_FWE_ < 0.05 are reported, with a cluster-defining voxel-wise threshold of P_FWE_ < 0.05 and minimal cluster size of k > 10. Results for the contrast “Stress Reactivity—stress induction vs. deep paced breathing” are reported with a cluster-defining voxel-wise threshold of P_FWE_ < 0.0001.

### Influence of HRV on whole-brain fMRI activation during the experimental task

Including a regressor for IBI values (re-sampled at the fMRI TR) in the individual GLM of participants, resulted in increased activation in a cluster encompassing the thalamus, putamen, anterior insula and medial cingulate cortex during the contrast “Stress Reactivity” (stress induction vs. deep-paced breathing, t = 5.21, *P* < 0.001, k = 2347, x = − 21, y = 26, x = 29, see Fig. 3e). Since we observed this result when comparing the original 1-level GLM with the GLM containing a regressor for IBI-values, increased activation indicates brain activation accounted for by the IBI regressor and therefore related to scan-by-scan variation in IBI values. To assess the specificity of this result, we built additional 1-level contrasts for the comparison of “stress induction” vs. baseline and “deep-paced breathing” vs. baseline. We then computed both contrasts with and without the inclusion of IBI-regressors. We found no differences for the contrast stress induction” vs. baseline but found patterns of activation during the contrast “deep-paced breathing” vs. baseline, similar to those observed during the contrast “Stress Reactivity” (bilateral thalamus, insula, medial cingulate cortex t = 5.17, *P* < 0.001, k = 1396, x = − 21, y = − 4, x = 50). We found no significant differences when comparing GLMs with and without the RMSSD regressor.

### Influence of stress and anxiety on whole-brain fMRI activation during the experimental task

**Fig. 4:**
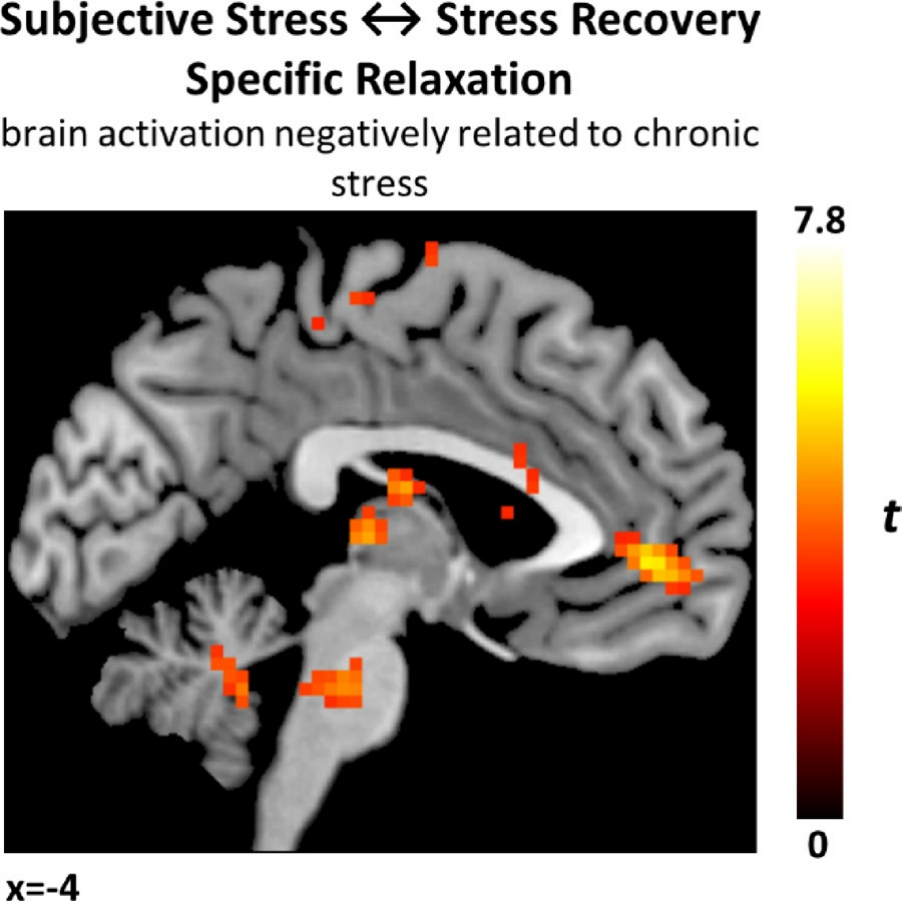
Whole brain regression with chronic stress. Significant negative association between brain activation during the contrast “Stress Recovery Specific Relaxation” (recovery deep-paced breathing vs. deep-paced breathing) and scores of the TICS-SCSS scale (chronic stress), significant activation in the anterior cingulate cortex/medial prefrontal cortex is shown. Results significant at a cluster-level FWE-corrected significance threshold of *P* < 0.05, with a cluster-defining voxel-wise threshold of *P* < 0.001 uncorrected and minimal cluster size k > 10.

We did not observe significant activation when including scores of the STAI (anxiety) and PSS (perceived stress) scales as regressors of interest in our statistical model. However, we found a significant negative association between brain activation during the contrast “Stress Recovery Specific Relaxation” (recovery deep-paced breathing vs. deep-paced breathing) and scores of the TICS-SCSS scale (chronic stress) in several regions, including the lobule IX of the cerebellum (t = 7.87, *P* = 0.01, k = 129, x = − 15, y = − 43, x = − 40), right frontal inferior operculum (t = 5.73, *P* < 0.001, k = 1014, x = 48, y = 14, x = 5), left insula (t = 6.59, *P* < 0.001, k = 2684, x = − 33,y = − 10, x = 20) and a cluster encompassing the anterior cingulate cortex/medial prefrontal cortex (t = 5.76, *P* = 0.011, k = 126, x = − 3, y = 47, x = − 1) See Fig. 4.

### Predictive relationship between brain activation and HRV: GAM analysis results

**Fig. 5:**
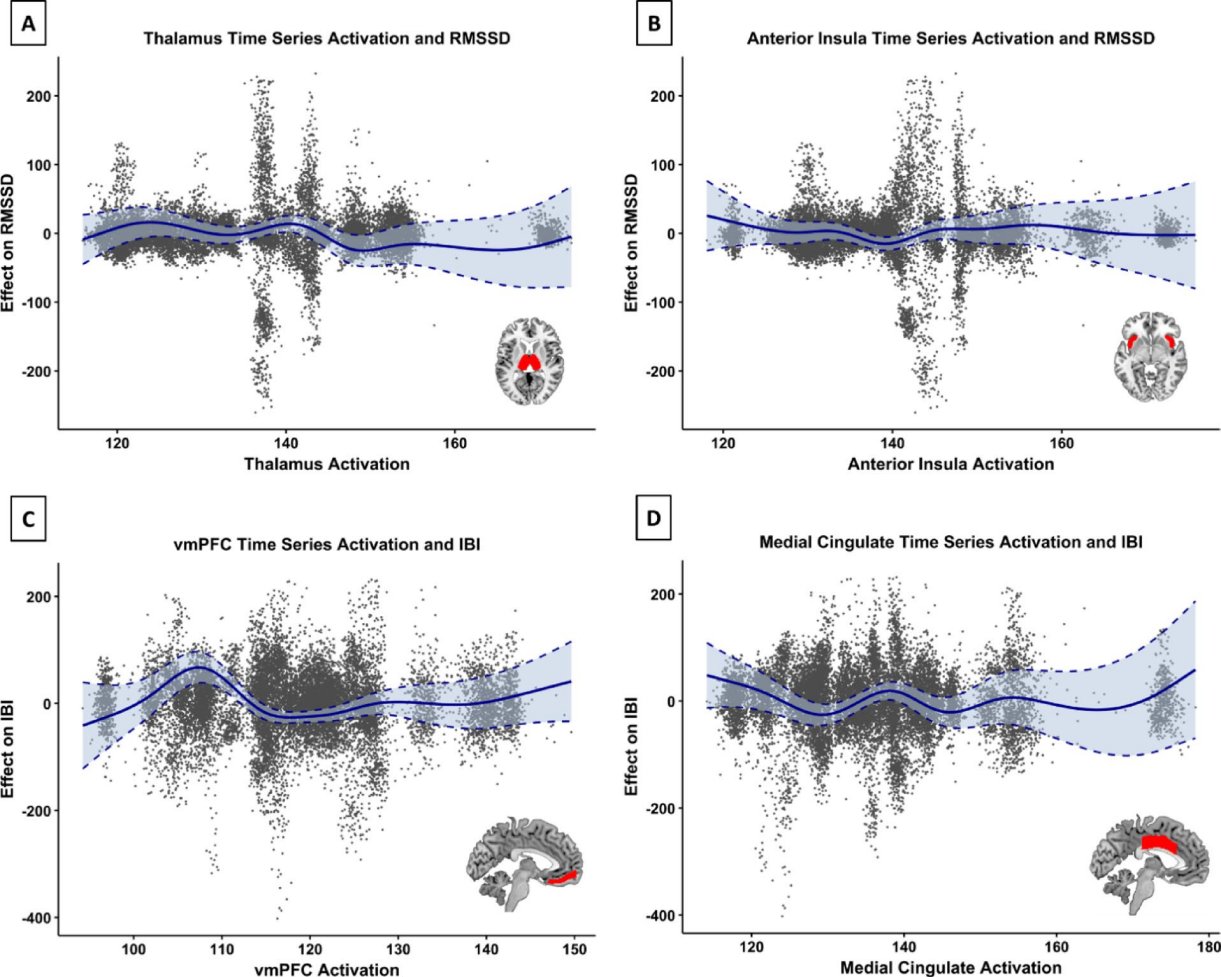
GAM results for the predictive relationship between brain activation and HRV. (**A**) Smooth function for thalamus time-series activation significantly predicts RMSSD (F = 4.155, *P* < 0.001, edf = 8.655). (**B**) Smooth function for anterior insula time-series activation significantly predicts RMSSD anterior insula (F = 3.121, P = 0.0024, edf = 7.991). (**C**) Smooth function for vmPFC time-series activation significantly predicts IBI (F = 8.770, P < 0.001, edf = 8.480), (**D**) Smooth function for medial cingulate time-series activation significantly predicts IBI (F = 5.755, P < 0.001, edf = 7.986). All models included subject and condition as fixed effects, and smooth terms were estimated using REML. The shaded band represents the 95% confidence interval around the smooth fit. The dashed lines outline the upper and lower bounds of the confidence interval. The gray dots represent the model residuals for each observation. Masks used to extract brain activation time series are shown at the lower right corner in each plot.

The GAM analysis revealed significant non-linear relationships between RMSSD and brain activation in the thalamus (F = 4.155, *P* < 0.001, edf = 8.655) and anterior insula (F = 3.121, *P* = 0.0024, edf = 7.991). In contrast, activation in the remaining brain regions (middle cingulate, anterior cingulate, amygdala, PAG and vmPFC) did not show significant relationships with RMSSD (*Ps* > 0.05). The overall model demonstrated good fit, explaining 67.6% of the deviance in RMSSD (adjusted R^2^ = 0.664, GCV = 1758.2). Furthermore, we found significant non-linear relationships between IBI and brain activation in the vmPFC (F = 8.770, *P* < 0.001), followed by the middle cingulate (F = 5.755, *P* < 0.001), anterior insula (F = 5.631,* P* < 0.001), thalamus (F = 4.380, *P* < 0.001), anterior cingulate (F = 2.670, *P* = 0.0038) and amygdala (F = 2.781, *P* = 0.0084). Activation in the PAG did not show a significant relationship with IBI (*P* > 0.05). The model demonstrated excellent fit, explaining 85.6% of the deviance in IBI (adjusted R^2^ = 0.851, GCV = 4708.8). All reported results were derived from GAMs with subject and condition covariates included as fixed effects, and model diagnostics confirmed the robustness of the fitted smooth terms. Full summary statistics are reported in Table 3, and visualizations of the smooth terms are provided in Fig. 5.

**Table 3:** Summary of GAM results for predicting RMSSD and IBI values based on brain activation.

Brain Region	edf	Ref.df	F-value	*P*-value
RMSSD				
Thalamus	8.655	8.961	4.155	< 0.001
Anterior Insula	7.991	8.765	3.121	0.0024
Middle Cingulate	1.030	1.059	1.098	0.3035
Anterior Cingulate	5.107	6.557	1.523	0.1817
Amygdala	3.584	4.737	0.584	0.5811
PAG	1.000	1.000	0.030	0.8631
vmPFC	4.046	5.288	1.751	0.1154
IBI				
Thalamus	8.271	8.876	4.380	< 0.001
Anterior Insula	8.677	8.970	5.631	< 0.001
Middle Cingulate	7.986	8.778	5.755	< 0.001
Anterior Cingulate	7.827	8.697	2.670	0.0038
Amygdala	8.241	8.873	2.781	0.0084
PAG	1.002	1.003	0.054	0.8192
vmPFC	8.480	8.933	8.770	< 0.001

edf, estimated degrees of freedom; Ref.df, reference degrees of freedom; PAG, periaqueductal gray; vmPFC, ventromedial prefrontal cortex. RMSSD Model fit: Adjusted R2 = 0.664, Deviance explained = 67.6%, GCV = 1758.2. IBI Model fit: Adjusted R2 = 0.851, Deviance explained = 85.6%, GCV = 4708.8.

To explore potential hemispheric differences in the relationship between insula activity and HRV, we repeated the GAM analyses with separate left and right anterior insula ROIs. For RMSSD, neither left nor right insula activation showed a significant association (left: F = 0.027, *P* = 0.87; right: F = 1.05, *P* = 0.40). However, for IBI, a pronounced hemispheric difference was observed: right insula activation showed a significant non-linear relationship with IBI (F = 10.73, *P* < 0.001), while the left insula did not (F = 1.51, *P* = 0.22).

### Granger causality analysis

We observed significant GC effects (Bonferroni-corrected empirical *P* < 0.05) from IBI to all seven ROIs. The corresponding F-values ranged from 4.03 (IBI to PAG) to 37.29 (IBI to anterior cingulate). In the reverse direction, all ROIs showed significant GC effects, with F-values ranging from 8.12 (PAG to IBI) to 49.49 (anterior cingulate to IBI). For RMSSD, significant GC effects were observed from RMSSD to five of the seven ROIs. The highest F-values were found for RMSSD to amygdala (5.69), vmPFC (6.71), and anterior cingulate (7.44). No significant effects were detected from RMSSD to mid-cingulate or thalamus. In the reverse direction, five ROIs showed significant GC effects, with F-values ranging from 4.82 (vmPFC to RMSSD) to 9.42 (amygdala to RMSSD). Mid-cingulate and PAG did not show statistically significant effects in this direction. A summary of the GC statistics for all tested directions is provided in Table 4.

**Table 4:** Bidirectional Granger causality statistics between heart rate variability (IBI, RMSSD) and brain regions.

		from			from						
		IBI			thalamus	ant_ins	mid_cing	ant_cing	amy	PAG	vmPFC
to	thalamus	26.67***	to	IBI	37.18***	47.68***	13.85***	49.49***	33.97***	8.12***	42.91***
	ant_ins	31.32***									
	mid_cing	11.92***									
	ant_cing	37.29***									
	my	15.82***									
	PAG	4.03*									
	vmPFC	33.27***									

		from			from						
		RMSSD			thalamus	ant_ins	mid_cing	ant_cing	amy	PAG	vmPFC
to	thalamus	3.34	to	RMSSD	5.71**	7.42***	2.75	3.65	9.42***	3.7	4.82*
	ant_ins	4.64*									
	mid_cing	3.64									
	ant_cing	7.44***									
	amy	5.69**									
	PAG	4.90*									
	vmPFC	6.71***									

Granger causality values between each pair of nodes (F-values). All reported effects were tested against a null distribution generated by 1,000 permutations of shuffled time series. *P < 0.05; **P < 0.005; ***P < 0.0005, Bonferroni corrected.

## Discussion

In this study, we examined the association between HRV and brain activation during stress regulation. We found that breathing-induced relaxation, as well as stress induction, had significant effects on IBIs but not RMSSD, and induced brain activation in CAN-related areas. HRV influenced CAN activity during stress induction and that chronic stress perception was related to reduced brain activation during post-stress relaxation. Finally, we showed that brain activation within the CAN can predict changes in HRV. This is the first study to investigate brain–heart interaction using a mixed-methods approach employing a comprehensive time-series analysis of both fMRI and HRV in connection with deep breathing, and may contribute to a deeper understanding of the role of stress reactivity and resilience on the central neural control of cardiovagal regulation.

In line with previous findings, breathing-induced relaxation as well as stress induction had a significant effect on IBI values, however, contrary to our expectations, we did not observe significant variation in RMSSD values across experimental conditions. A negative relationship between perceived stress and IBI variation was observed during post-stress deep breathing, indicating higher stress linked to lower heart rate variation during recovery. These findings support research indicating that stress may blunt autonomic flexibility^59–61^, for example through increased sympathetic output effected by regions such as the insula and ACC. This, in part, is achieved through increased blood flow in CAN regions due to neurovascular coupling of the amygdala during stress with higher order brain centers such as the ACC, PFC, insula and putamen^2,62^. Increased activation of these regions, in turn, leads to increased peripheral vasoconstriction^1^, allowing for less variability.

During slow-paced breathing, we observed significant activation in areas of the CAN, such as the bilateral postcentral gyrus, inferior frontal gyrus, thalamus, and medial cingulate gyrus, consistent with previous research demonstrating enhanced CAN activity at slow breathing rates of ~ 5.5 breaths/min^13^. This supports the notion that deep breathing engages sensory and cognitive control networks, potentially enhancing mindfulness and self-regulation through respiratory driven brain oscillations^29,63^. In contrast, the transition from deep breathing to stress induction was linked to increased activation in brain areas commonly associated with emotional processing, threat response and autonomic regulation, aligning with previous stress-induction studies^39,64^. The ACC has been found to mediate sympathetic modulation of heart rate during stress induction^2,9^, while the PAG integrates autonomic control with behavioral responses to stress^24^ and cardiorespiratory responses^65^. On the other hand, the superior medial frontal gyrus is implicated in self-referential processing, executive functioning and emotion regulation mainly via the parasympathetic nervous system^66^. Increased activity, as shown in our findings, could indicate a medial prefrontal mediated down-regulation of the sympathetic stress response. However, in coronary artery disease, higher activation in medial prefrontal regions has been associated with increased risk of major adverse cardiovascular events^67^, suggesting differing implications of stress reactivity in healthy versus disease states. During recovery from stress, activation was observed primarily in the middle temporal gyrus, an important component of the default mode network^68^. Since activation of the DMN is negatively correlated with sympathetic activation^69^, our finding could be suggestive of engagement of higher cognitive processing, self-referential processing and emotional regulation to accommodate stress recovery^70^. Lastly, during breathing-induced recovery from stress induction, we found activation in brain regions associated with sensory and emotional information integration. These findings are similar to slow-breathing before stress-induction, with a distinct overlap within the insula, which is in line with previous studies and underlines the central role of the insula in tonic inhibition of the sympathetic efferent neural tone^11,71,72^.

We also observed that higher perceived chronic stress correlated with reduced CAN activation during post-stress relaxation, particularly in the cerebellum, frontal operculum, left insula, mPFC, and ACC. This supports findings that autonomic control regions are implicated in regulation of the hypothalamus-pituitary axis (HPA), which regulates cortisol secretion^40^. In chronic stress, due to excessive cortisol levels, negative feedback effects likely blunt activity and reactivity in the higher cognitive centers modulating the HPA^73^. Furthermore, high glucocorticoid levels may disrupt CAN activation through neurotoxic effects, particularly in the amygdala and prefrontal cortex, given their high glucocorticoid receptor density^74^. This suggests that chronic stress may impair the neural mechanisms involved in stress recovery, blunting the reestablishment of parasympathetic control through maladaptive bottom-up feedback to autonomic brain regions as well as potential neurotoxic effects.

We found that when comparing stress induction to breathing-induced relaxation, scan-by-scan variations in IBI were associated with activation in CAN regions such as the thalamus, putamen, anterior insula and medial cingulate cortex. Although it is still a topic of discussion whether this coupling is in the context of bottom-up or top-down regulation, a dynamic interplay is assumed^6,75^. Specifically, ascending interoceptive pathways from the heart to higher cognitive centers have been identified as major effectors of top-down adjustments of autonomic regulation^76^. Our results are in line with previous observations of shared neural substrates across sympathetic and parasympathetic autonomic regulation, especially in regards to the dual autonomic role of the insula^77^ and the function of the thalamus as an autonomic control center^78^. The insula is assumed to have a split role in autonomic control, with hemispherical lateralization of autonomic effect, explaining the dual activation during both stress and relaxation phases^71^. We were able to demonstrate such a hemispheric difference in relation to IBI, as right insula activation showed a significant non-linear relationship with IBI, while the left insula did not. These results suggest that the right insula plays a predominant role in modulating IBI in the context of our experimental paradigm, in line with previous reports of hemispheric lateralization of insular contributions to autonomic control^71^. On the other hand, the thalamus acts as a crucial pathway between the amygdala and mPFC and dynamically modulates autonomic output based on demand, such as increased sympathetic outflow to accommodate a stress response and reestablishing parasympathetic influence in the recovery process^78^. As HRV increased, there was a stronger coupling between the dorsal ACC/amygdala with the brainstem, thalamus, putamen and dorsolateral prefrontal cortex. Striatal regions, such as the putamen and caudate, are also involved in the stress response^79^ and are known to mediate cognition and stimulus response learning^80^. They show close functional connections to the mPFC and amygdala, with increased functional connectivity during phases of higher HRV^81^. Overall activity in the putamen and caudate has been shown to be enhanced during the stress response^82^. Taken together, our results suggest a tight coupling between cardiovascular and neural responses to stress.

Finally, we found non-linear predictive relationships between CAN activation and HRV throughout the different experimental conditions. Specifically, we found that brain activation in the thalamus and anterior insula were correlated with RMSSD variations, whereas variations in IBI were significantly associated with activity in multiple regions of the CAN, including the vmPFC, middle cingulate cortex, and amygdala, as well as the anterior insula and thalamus. The thalamus, as a sensory relay hub, may facilitate the dynamic adjustment of autonomic responses based on sensory input, while the anterior insula, known for its involvement in interoception, could mediate the subjective experience of these physiological states^83,84^. Similarly, the strong relationships between inter-beat interval (IBI) and brain activation in the ventromedial prefrontal cortex (vmPFC), middle cingulate, anterior insula, and thalamus highlight their broader involvement in regulating heart rhythm^66^. The amygdala is central to both the initiation and regulation of autonomic responses, particularly in relation to HRV, as well as being a key node in activating the HPA axis during stress^85^. Higher HRV is positively correlated with functional connectivity between the amygdala and regulatory regions such as the dorsal ACC, thalamus, and prefrontal cortex^56,75^. The amygdala relays information to higher cortical centers, namely the prefrontal cortex and ACC which in turn exert top-down inhibitory control^66,86^. This functional connectivity varies by age and sex^87,88^, but overall aligns with the neurovisceral integration model, associating higher HRV with more adaptable regulatory networks, supported by amygdala-prefrontal interactions. The model’s high explanatory power (85.6% deviance explained for IBI) underscores the robustness of these relationships and suggests that neural activity can be a reliable predictor of specific HRV components. In our Granger causality analysis, we were further able to demonstrate strong bidirectional links between the brain and the heart. For IBI, significant effects of IBI on all seven regions of interest as well as vice versa were observed, suggesting predictive effects of both bottom-up and top-down regulation. In terms of the bottom-up perspective for RMSSD, activation in the amygdala and ACC were correlated with RMSSD variation, whereas in the top-down direction, particularly the vmPFC and amygdala showed influence of RMSSD values. These results provide compelling evidence for a bidirectional predictive relationship between brain activation and HRV, expanding existing literature on brain–heart interactions^6,8,52,75^, shedding light on the neural mechanisms underpinning autonomic regulation and reinforcing the utility of HRV as a biomarker for understanding the interplay between central and peripheral systems.

### Limitations

While our study provides important new insight into the brain–heart axis, several limitations need to be addressed. Our study focused on healthy young adults, limiting generalizability. While prior research on HRV impairment has examined affective and cardiovascular disorders^89,90^, our study assessed only short-term stress in healthy individuals. Furthermore, HRV measures over extended periods (e.g., 24 h) improve accuracy^44^. Accordingly, our timeframe may have been insufficient to detect RMSSD changes. In this context, the non-significant effect observed is likely attributable to methodological constraints, such as the use of a PPG signal rather than an ECG, or the relatively short recording period. Additionally, the use of an arithmetic stress task within the MRI environment may not have elicited a sufficiently robust stress response, or participants may not have achieved adequate relaxation, resulting in a suboptimal parasympathetic baseline. These factors may also account for the observed trend in RMSSD changes despite the lack of statistical significance. While psychosocial stress tasks, such as the MIST, are widely used in research for their simplicity and reliability, they may induce weaker stress responses compared to physiological stressors (e.g. pain), which might activate the HPA axis more robustly^91^. The exploratory correlations between HRV indices and psychometric scores should be interpreted with caution, as they were not corrected for multiple comparisons and may reflect chance findings. Future studies with larger samples and hypothesis-driven analyses are needed to confirm these relationships. Furthermore, PPG-based HRV measurements are susceptible to respiratory artifacts, whereas ECG recordings provide higher accuracy^92,93^. Although previous studies have reported hemispheric lateralization in the autonomic functions of the insula, we used bilateral ROIs and did not assess hemispheric differences in the current analysis. Future studies with lateralized ROI definitions and sufficient statistical power may help clarify whether stress- and relaxation-related insula activation reflect functionally distinct hemispheric contributions. Additionally, the use of a lenient high-pass filter of 480 s may have retained slow-frequency signal drifts, which could confound the interpretation of low-frequency effects observed in the data. Finally, the methods employed in this study do not allow a direct assessment of causality. While the use of GAMs allows exploring directional associations, these findings remain correlational. Future studies should incorporate experimental manipulations or advanced modeling approaches to establish causal links between neural and autonomic activity.

## Conclusion

Taken together, our study supports previous findings regarding the brain–heart axis and provides new insights into the complex relationship between HRV and brain regions involved in autonomic regulation. Consistent with previous research, our observations underline the central role of the CAN in mediating stress-related cardiovascular responses. Our findings show that in a healthy organism, autonomic flexibility can be tracked by HRV and fMRI across states of relaxation and stress. Furthermore, we showed that the impact of chronic stress on autonomic control may be regulated through reduced activation in the insula and mPFC during recovery from stress. Future investigations should further investigate the brain–heart axis and its role in health and disease, which could provide new avenues for clinical and therapeutic interventions targeted at stress resilience and improved autonomic regulation.

## Data Availability

The data underlying this article will be shared on reasonable request to the corresponding author.
